# Cholesteatoma surgery in the pediatric population: remaining challenges in the era of mastoid obliteration

**DOI:** 10.1007/s00405-022-07669-0

**Published:** 2022-10-08

**Authors:** Victor J. Kroon, Steven W. Mes, Pepijn. A. Borggreven, Rick van de Langenberg, David R. Colnot, Jasper J. Quak

**Affiliations:** 1 Department of Otolaryngology and Head and Neck Surgery, Diakonessenhuis Utrecht, Bosboomstraat 1, 3582 KE Utrecht, The Netherlands; 2 Faculty of Medicine, University Utrecht, Utrecht, The Netherlands; 3 Otolaryngology-Head and Neck Surgery, Amsterdam UMC Location Vrije Universiteit Amsterdam, De Boelelaan 1117, Amsterdam, Netherlands

**Keywords:** Cholesteatoma, Mastoid, Pediatric, S53P4, Obliteration

## Abstract

**Purpose:**

To present the first pediatric study on the safety and efficacy of mastoid obliteration using S53P4 bioactive glass (BAG) for cholesteatoma surgery.

**Methods:**

A single-center retrospective cohort study was conducted. Inclusion criteria were pediatric cases (≤ 18 years) and at least at least one year of follow-up including non-echo planar diffusion-weighted MRI to assess cholesteatoma recidivism. Both canal wall up (CWU) and canal wall down (CWD) procedures were evaluated.

**Results:**

A total of 61 cases (56 patients) were included. Most cases had an otologic history before the development of the cholesteatoma. CWU procedure was performed in 18 cases (30%) and CWD procedure in 43 cases (70%). The cholesteatoma recidivism rate was 33% after a mean follow-up period of 58 months. Kaplan–Meier curve estimated a 5-year recidivism rate of 40%. Few complications were seen that were all minor and resolved spontaneously or after local or systemic treatment. Control of the infection (merchant grade 0–1) was achieved in 98% of the cases. Closure of the air–bone gap within 20 dB was achieved in 22% of the cases with complete audiometric evaluation.

**Conclusion:**

In this MRI-controlled study, we show the safety and efficacy of S53P4 BAG for mastoid obliteration in a pediatric cholesteatoma cohort. Postoperative complications were both rare and minor, and a dry ear was achieved in almost all patients. Nevertheless, persistent hearing loss and the apparent high recidivism rate reflect the challenging nature of pediatric cholesteatoma.

**Supplementary Information:**

The online version contains supplementary material available at 10.1007/s00405-022-07669-0.

## Introduction

Pediatric cholesteatomas remain a challenge for clinicians. The disease tends to be more aggressive in children when compared to adults, with higher rates of recidivism and subsequent revision surgeries [1, 2]. Obliteration of the mastoid after either canal wall up (CWU + MO) or canal wall down (CWD + MO) procedure has recently gained interest, showing equal and potentially lower recidivism rates when compared to non-obliterative techniques [3, 4]. This is most likely the result of multiple factors. First, obliteration creates a physical barrier to prevent recurrent retraction pockets. Second, reduction in the mucosal surface due to obliteration of the mastoid results in a reduction of gas absorption and, therefore, normalization of middle ear pressure [5]. Third, the CWD + MO technique allows for surgeons to utilize the advantages of CWD, i.e. better overview and accessibility, without the potential disadvantages of a cavity, such as the necessity of regular cleaning and the higher rates of otorrhea.

Traditionally, cholesteatoma surgery was staged between primary surgery that was focused on eradication of the disease and secondary surgery, also called second look surgery. The latter was mainly intended for the evaluation of recidivism. However, the development of the non-echo planar diffusion-weighted magnetic resonance imaging (non-EP DWI MRI) allows for a highly sensitive and specific non-invasive assessment of cholesteatoma recidivism, also in pediatric cases [6–8].

Many different materials can be used for obliteration of the mastoid, including autologous and synthetic materials. In 2011, our department started using S53P4 bioactive glass (BAG) for mastoid obliteration. S53P4 BAG has two unique qualities, namely, inhibition of bacteria and the stimulation of bone growth with osteostimulation and osteoconduction. A rise in pH due to ion release in combination with an increase in osmotic pressure results in an inhospitable environment for bacteria, which has been confirmed in vitro on a range of clinically relevant bacteria [9, 10]. Furthermore, a recent in vitro study has shown that the keratinocyte growth is inhibited by the rise in pH, possibly preventing cholesteatoma development [11]. The osteostimulative and osteoconductive properties are related to the formation of a scaffolding layer for the osteoblasts [12, 13]. Other important qualities of S53P4 BAG are the unlimited availability, no need for impregnation with antibiotics, preservation of volume over time and no donor site morbidity [14–16].

Earlier studies on S53P4 BAG in cholesteatoma surgery have shown promising results [17, 18]. However, few studies on mastoid obliteration in a pediatric population with MRI follow-up have been conducted in general. So far, no studies have been published on S53P4 BAG for mastoid obliteration in pediatric cholesteatoma surgery. Therefore, we present the first study on the safety and efficacy of S53P4 BAG in a pediatric cohort, with long-term MRI controlled follow-up.

## Materials and methods

### Ethical considerations

This retrospective cohort study was conducted at a secondary referral center in the Netherlands. The study was in accordance with the ethical standards of the hospital institutional review board (registration number W21.162) and the 1964 declaration of Helsinki. Formal consent is not required for this type of retrospective study. The BAG S53P4, produced by BonAlive Biomaterials Ltd. (Turku, Finland), has received clearance for clinical use by the CE (2004).

### Patient characteristics

Eligible for inclusion were all pediatric patients (≤ 18 years, range was 5–18 years) with cholesteatoma who underwent primary or revision single stage tympanomastoidectomy with mastoid obliteration using S53P4 bioactive glass in the period 2011–December 2020. Cholesteatoma was defined as a mass formed by keratinizing squamous epithelium in the tympanic or mastoid area, in accordance with the EAONO/JOS Joint Consensus Statement by Yung et al. [19]. Keratin masses presenting solely in a former radical cavity and that could relieve spontaneously or by cleaning at the outpatient clinic were not considered cholesteatoma. Other inclusion criteria were a minimum follow-up period of one year postoperatively and the use of non-EP DWI MRI to evaluate recidivism. Patients were excluded if no non-EP DWI MRI was performed postoperatively and/or if previous obliteration had been performed. Ear-related problems that presented > 1 year before diagnosing the cholesteatoma were extracted from the patient file and included atelectasis, otorrhea, previous ear tubes or paracentesis, previous tympanoplasty and cholesteatoma. In patients with bilateral cholesteatomas, ear-related problems > 1 year before diagnosis were reported separately for each ear.

### Surgical technique and follow-up

Both canal wall up (CWU + MO) and canal wall down (CWD + MO) tympanomastoidectomy were evaluated, the surgical approach chosen depended on the surgeon’s assessment. If the operator felt confident that it was possible to remove the cholesteatoma in total without the removing the posterior wall, a CWU + MO procedure was performed. If, however, this was not possible then the posterior wall was removed for improved visualization. Under general anesthesia, a retro-auricular skin incision was made to perform an extensive mastoidectomy. All cholesteatoma was subsequently removed from the middle ear and mastoid. Reconstruction of ossicular chain was performed if considered feasible. Intraoperative nerve monitoring was used to identify, confirm and monitor the facial nerve. In CWU + MO procedure, the posterior wall of the external ear canal was preserved and the antrum was closed with tragus or conchal cartilage or, if the malleus head and incus were removed, cartilage was placed at the border of the meso- and epitympanum. In CWD + MO procedures, the posterior wall of the external ear canal was reconstructed with cartilage. The mastoid cavity and, in case of removal of the malleus head and incus or in case of a CWD + MO procedure, the epitympanum, was obliterated with S53P4 BAG granules moistened with 0.9% sterile saline solution. Fibrine glue (Tissucol, produced by Baxter, Deerfield, IL, USA) was applied in the first years to stabilize the BAG granules but later abandoned as it showed no additional benefit. Musculoperiosteal vascularized flaps were used to cover and close the obliterated cavity. Current practice in our clinic in both CWU + MO and CWD + MO procedures is to administer 30 mg/kg (maximum of 2000 mg) cefazolin i.v. intraoperatively and additionally, only after CWU + MO procedures, a passive drain in the obliterated cavity and amoxicillin/clavulanic acid 3–5 days postoperatively. Follow-up consisted of clinical otoscopy at 1 and 8 weeks following surgery, at 3–6 month intervals for 1 year and thereafter once yearly unless more frequent visits were deemed necessary. To objectively assess cholesteatoma recidivism, a non-EP DWI MRI was made at 1, 3 and 5 years following surgery, or more frequent if deemed necessary by the treating physician. MR imaging was performed on a 1.5 T MR imaging scanner (Avanto, Siemens, Erlangen, Germany) with a 12 element head-matrix-coil with 12 pre-amplifiers. T2 HASTE coronal images were acquired using the following settings: TR/TE = 1400/109 ms, flip angle = 140°, slice thickness = 3 mm, Matrix = 192 × 192, b factors = 0 and 1000 mm^2^/s, acquisition time = 5 min 40 s. The apparent diffusion coefficient (ADC) was calculated from the *b* = 0 and *b* = 1000 images.

### Outcome measures

The following outcome parameters were included: procedure safety, Merchant grade at the most recent out-patient visit, cholesteatoma recidivism and audiometric performance. Procedure safety was defined as the absence of peri- and post-operative complications up to six months following surgery. Merchant’s grading system was used to evaluate postoperative otorrhea [20]. Grades 0–1 were defined as control of infection and grades 2–3 as no control of the infection. Cholesteatoma recidivism was defined as a mass of keratinizing squamous epithelium invading the middle ear diagnosed by either micro-otoscopy or non-EP DWI MRI. Cholesteatoma recidivism consisted of both recurrent and residual cholesteatoma. Residual disease was defined as cholesteatoma left behind inadvertently during the first surgery, recurrent as the development of a new retraction pocket with retention of keratinizing squamous epithelium. It is also important to report the recurrence and residual rate separately, as they are different entities [19]. Audiometric evaluation was performed pre- and postoperatively using pure-tone audiometry at 500, 1000, 2000, 4000 Hz for both air and bone conduction (AC and BC, respectively) and the average AC and BC were calculated. The average air–bone gap (ABG) was calculated from the difference in AC and BC. Audiometric data were only analyzed if both pre- and postoperative hearing test were complete and performed within 6 months before and after surgery.

### Statistical methods and data analysis

Data were analyzed using SPSS statistics (version 27, IBM Corp, Armonk, NY, USA). Continuous data were presented as mean ± standard deviation (S.D.) or median with interquartile range (IQR), depending on normal or non-normal distribution. Kaplan–Meier curve was used to extrapolate the 5-year recidivism rate, compensating for loss to follow-up and patients with a follow-up of less than 5 years. Fisher’s exact test, Chi-squared test and Mann–Whitney *U* test were used for univariate analysis to determine predictive factors for cholesteatoma recidivism. For audiometric evaluation, Wilcoxon signed-rank test was used for comparison of pre- and postoperative AC, BC and ABG. Mann–Whitney *U* test was used to compare postoperative ABG between different subsets of cases. *p* value ≤ 0.05 was considered statically significant.

## Results

We have performed a total of 789 mastoid obliterations using S53P4 BAG in our hospital, of which 167 were pediatric cases. Of these, 65 surgeries were to treat cholesteatoma, al being performed in the period of 2011–December 2020. Four cases did not have adequate follow-up with non-EP DWI MRI: in three cases the MRI is planned but not yet performed at the time of publication and one case was still in follow-up but did not receive an MRI scan. In total, 61 cases (56 patients) were included of which 29 were left ears (Table 1). Five patients were operated on both ears. Thirty-nine (64%) of the cases were male and the mean age at time of surgery was 11 years (S.D. 4, range 5–18). Of the included cases, 18 (30%) were previously operated for cholesteatoma, of which three cases twice and two cases three times.

**Table 1:** Patient characteristics and surgical technique, *n* (%)

Total cases included	61 (100)
Male	39 (64)
Age in years (mean ± SD)	11 ± 4
Primary surgery	43 (70)
Side	
Left	29 (48)
Right	32 (52)
Location cholesteatoma	
Pars tensa	21 (34)
Pars flaccida	23 (38)
Combination tensa and flaccida	13 (21)
Residu previous surgery	3 (5)
Unknown	1 (2)
Type of surgery	
CWU	18 (30)
CWD	43 (70)
Ossicular chain reconstruction	
Intact	6 (10)
Previously placed PORP left in place	2 (3)
Cartilage and/or fascia	10 (16)
Incus interposition	2 (3)
PORP	22 (36)
TORP	17 (28)
No reconstruction	2 (3)

*CWU* canal wall up, *CWD* canal wall down, *PORP* partial ossicular replacement prosthesis, *TORP* total ossicular replacement prosthesis

### Otologic history

Most patients (55 cases, 90%) had a history of ear problems before the development of the cholesteatoma, being under control currently or previously at the general practitioner or our hospital. Most frequently seen were tympanotomy tubes (42 cases, 69%), followed by otorrhea (39 cases, 64%), atelectasis (28 cases, 46%), tympanic membrane perforations (9 cases, 15%) and paracentesis (6 cases, 10%). Interestingly, 37 cases (61%) were under regular follow-up (i.e. at least every 6–9 months) at our ENT department for ear problems before they developed cholesteatoma. Nine of the cases with atelectasis had previously undergone surgery for cartilage reinforcement of the tympanic membrane.

### Surgical technique and safety

Patients were operated by four different otologists in our department. CWU + MO procedures were performed in 18 cases (30%) and CWD + MO procedures in 43 cases (70%). Four of the CWU + MO procedures and 14 of the CWD + MO procedures had previously been operated for cholesteatoma (22% and 33%, respectively).

The ossicular chain was reconstructed in 51 cases: 10 cases (16%) with cartilage and/or fascia, 2 cases (3%) with incus interposition, 22 cases (36%) with a titanium partial ossicular replacement prosthesis and 17 cases (28%) with a titanium total ossicular replacement prosthesis. In the remaining 10 cases, a previously placed PORP was left in place (2 cases, 3%) or the ossicular chain was either left intact (6 cases, 10%) or not restored during the initial surgery (2 cases, 3%).

Major complications such as liquor leakage, damage to the facial nerve or total deafness were not observed. Minor postoperative complications were observed in 7 cases (11%), al resolving spontaneously or after local or systemic treatment. The minor complications included retro-auricular wound infection requiring oral antibiotics (*n* = 2), mild otorrhea requiring oral antibiotics (*n* = 2) and recurrent perforation of the tympanic membrane (n = 3).

### Cholesteatoma recidivism

**Fig. 1:**
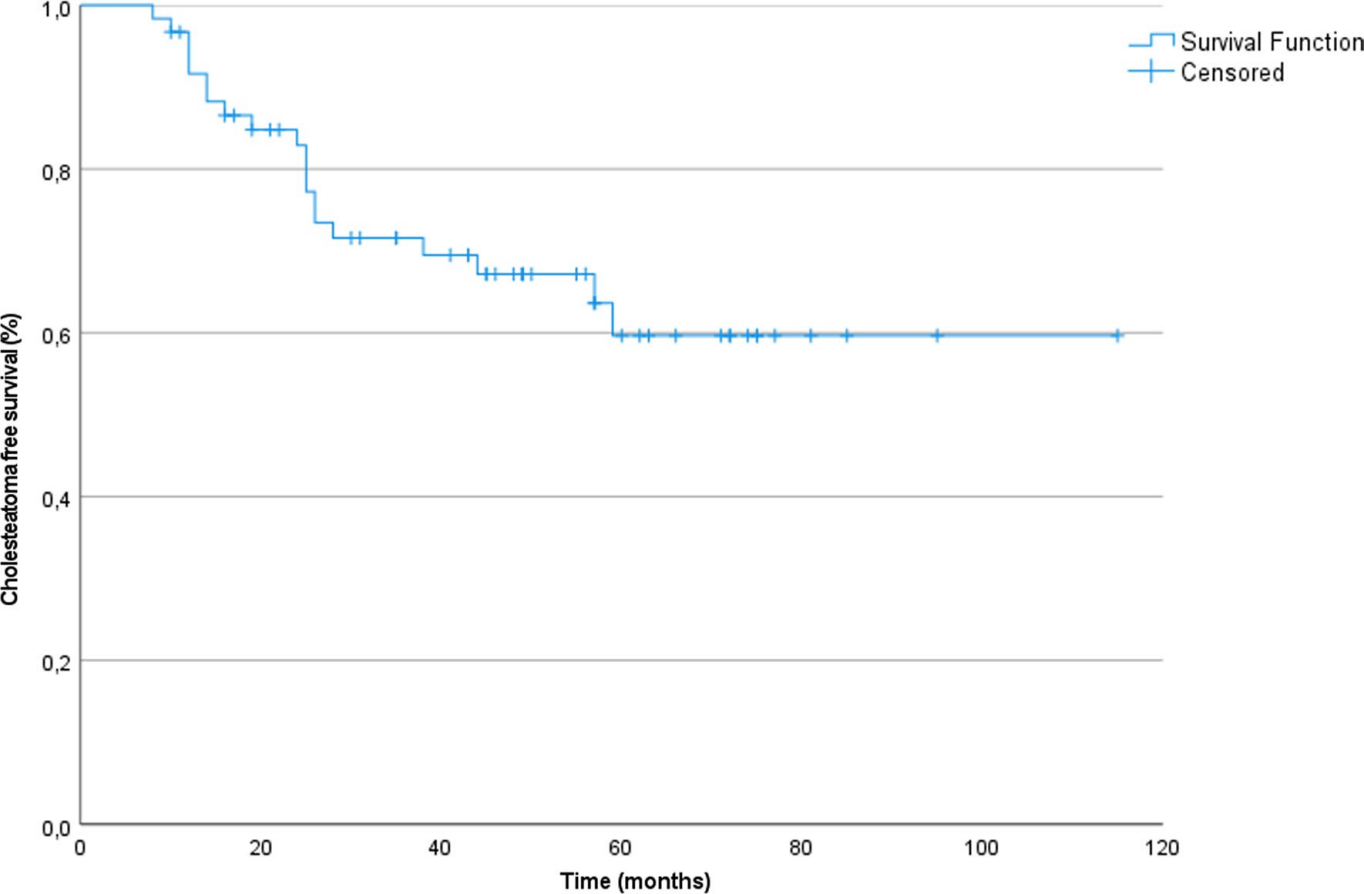
Kaplan–Meier curve to estimate cholesteatoma free survival, indicating 40% recidivism rate at 5 years following surgery

The mean follow-up period was 58 months (S.D. 26, range 12–123) with a mean follow-up period by MRI of 50 months (S.D. 25, range 12–122). All cases were followed-up by non-EP DWI MRI and a total of 161 scans were made, averaging at 2.6 per case. Recidivism rate was 33% (20 cases) with a mean time to event of 24.7 months (S.D. 14.3, range 8–59). The Kaplan–Meier curve, used to extrapolate the recidivism rate while compensating for follow-up time, estimated a 5-year recidivism rate of 40% (Fig. 1). Eight recidivisms were recurrent cholesteatomas and twelve were residual cholesteatoma. After CWU + MO, the residual rate was 17% (3/18) and the recurrence rate was 0%. After CWD + MO, the residual rate was 21% (9/43) and the recurrent rate was 19% (8/43). Specific information on the recurrent or residual cases is presented in Supplementary Table 1. When solely looking at primary cholesteatomas, 16/43 patients (37%) developed a recidivism, six being recurrent and ten being residual. The recidivisms were detected by either non-EP DWI MRI (*n* = 17, 85%) or otoscopic examination (*n* = 3, 15%) (Supplementary Table 1). All of the residual cholesteatomas and 5/8 (63%) of the recurrent cholesteatomas were detected by MRI. The average size of the cholesteatoma recidivism on the MRI was 8.4 mm (S.D. 4.7 range 3–23). Recidivisms were located in the epitympanum (*n* = 11), sinus tympani (*n* = 4), stapes footplate (*n* = 3), mastoid (*n* = 1) and mesotympanum (*n* = 1). Specific information on the recurrent or residual cases is presented in Supplementary Table 1. No cholesteatoma involving the obliterated cavity was observed. However, we did observe one recurrent cholesteatoma in the mastoid, but this developed in an area with insufficient obliteration after loss of BAG during healing. Recidivism rates per surgeon ranged from 23% to 45%. Five cholesteatomas were discovered in the first year following surgery, five in the second year, six in the third year, two in the fourth year and two in the fifth year postoperatively (Supplementary Table 1).

Surgical techniques to remove the recidivism were either (endoscopic) transcanal (5/20, 25%), CWU + MO (1/20, 5%), or CWD + MO (14/20, 70%). No difficulties were experienced when removing the BAG for access to the middle ear during revision surgery, regardless of the time since the initial surgery. Four of the recidivism cases (20%, three residual cholesteatomas and one recurrent cholesteatoma) developed a second recidivism after revision surgery, all requiring a CWD + MO procedure. The remaining 16 cases were cholesteatoma free with a mean follow-up period of 30 months (S.D. 21, range 0–60).

### Analysis of factors associated with cholesteatoma recidivism

Analysis of factors possibly associated with the development of recurrent or residual cholesteatoma was performed. A history of otorrhea prior to the development of the cholesteatoma was significantly associated with residual cholesteatoma (11/12 vs 21/41, *p* = 0.017; Table 2). Interestingly, age at time of the primary surgery could not be associated with residual or recurrent cholesteatoma (11 years vs 12 years, *p* = 0.924 and 11 years vs 12 years, *p* = 0.775, respectively, Table 2). All other evaluated factors are presented in Table 2.

**Table 2:** Analyses of otologic history before cholesteatoma development for factors associated with residual and recurrent cholesteatoma (*n*)

	Residual cholesteatoma		*p* value	Recurrent cholesteatoma		*p* value
	Yes (*n* = 12)	No (*n* = 41)		Yes (*n* = 8)	No (*n* = 41)	
Age in years (median IQR)	11 (8–15)	12 (8–15)	0.924	11 (8–13)	12 (8–15)	0.775
CWU procedure	3	15	0.507	0	15	0.044*
Location cholesteatoma						
Pars Tensa	4	13	0.838	4	13	0.432
Pars flaccida	5	15	0.838	3	15	1.000
Ossicular chain reconstruction						
PORP	3	14	0.711	5	14	0.233
TORP	3	12	0.711	1	12	0.425
History of ear problems in general	11	37	1.000	7	37	1.000
Otorrhea	11	21	0.017*	7	21	0.115
Perforation of the tympanic drum	2	7	1.000	0	7	0.581
Paracentesis	1	4	1.000	1	4	1.000
Tympanotomy tubes	5	31	0.038*	6	31	1.000
Atelectasis	2	21	0.048*	5	21	0.706
Cartilage reinforcement	0	7	0.329	2	7	0.628
Previous cholesteatoma	2	14	0.307	2	14	1.000
Under control at our department	3	28	0.017	6	28	1.000

^*^Significant using Fisher’s exact test

### Merchant grade

Preoperatively, 35/61 cases (57%) had a merchant grade of 2–3. When evaluating the Merchant grade at one year postoperatively, 54/61 cases (89%) had control of the infection (merchant grades of 0–1). Of the seven cases with a merchant grades of 2–3, four had cholesteatoma recidivism as cause of the otorrhea. At the most recent out-patients visit, merchant grades 0–1 was achieved in 60 cases (98%, 50 cases grade 0, 10 cases grade 1). Only one patient had a merchant grade of 2, no persistently wet ears (merchant grade 3) were observed.

### Audiometric evaluation

**Fig. 2:**
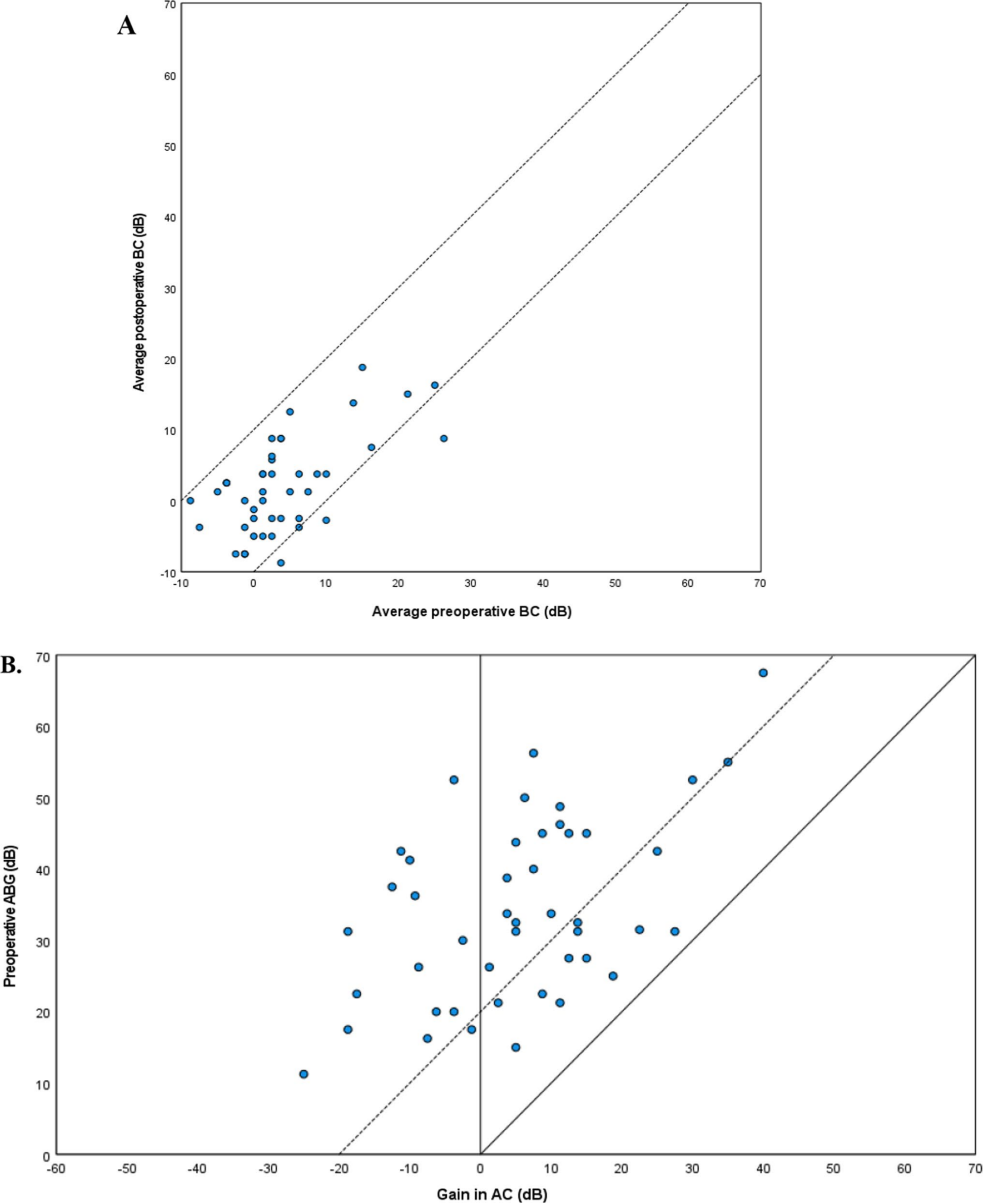
Amsterdam Hearing Evaluation Plots (AHEP) of all cases with complete audiometric evaluation. A. Preoperative BC plotted against postoperative BC. Dotes within the 2 diagonal lines represent cases of whom the BC did not change more than 10 dB. B. Postoperative gain in AC plotted against the preoperative ABG. The solid diagonal line indicates total closure of the preoperative ABG, the dotted diagonal line indicates closure of the preoperative ABG to 20 dB

Complete audiometric evaluation was available in 46 cases (75%). Reasons for incomplete audiometric evaluation were missing frequencies (*n* = 9) or audiometric evaluation performed more than six months before or after surgery (*n* = 6). The average AC improved significantly pre- to postoperative (35.0 dB IQR 27.5–47.8 vs 30.6 dB IQR 20.9–46.3, *p* = 0.014, Table 3). However, since the gain was only 4.4 dB, the clinical relevancy might be limited. The average BC did not change significantly, thus showing no sign of inner ear damage due to the surgery or obliteration material (2.5 dB vs 1.3 dB, *p* = 0.060). The average ABG also did not change significantly (32.5 dB vs 28.8 dB, *p* = 0.104). When analyzing the subset of patients with a TORP or PORP, a significant change in average ABG was seen pre- to postoperatively (33.8 dB vs 26.3 dB, *p* = 0.003) This effect was more pronounced in the subset of patients receiving a TORP (45.0 dB vs 28.8 dB, *p* = 0.005) and not significant in the subset of patients receiving a PORP (31.3 dB vs 25.0 dB, *p* = 0.178). However, when comparing the postoperative ABG between the PORP and TORP groups, no significant difference could be detected (25.0 dB vs 28.8 dB, *p* = 0.370). The postoperative ABG was not significantly different between the CWU + MO and CWD + MO group (26.9 vs 28.8, *p* = 0.310). ABG closure, i.e. ABG ≤ 20 dB was achieved in 10 patients (22%) postoperatively (Fig. 2).

**Table 3:** Audiometric evaluation for all patients with complete audiometry (Median, IQR)

	Preoperative	Postoperative	Gain	*p* value
Bone conduction	2.5 (− 1.3 to 6.3)	1.3 (− 2.5 to 6.3)	1.2	0.060
Air conduction	35.0 (27.5 to 47.8)	30.6 (20.9 to 46.3)	4.4	0.014*
ABG	32.5 (24.4 to 45.0)	28.8 (21.6 to 41.3)	3.7	0.104

Values are in dB

IQR, Interquartile range; ABG, Air–bone gap;

*p values ≤ 0.05 using Wilcoxon signed rank test

## Discussion

In this retrospective cohort study, we showed that mastoid obliteration using S53P4 BAG is a safe technique in pediatric cholesteatoma surgery. Very few complications were seen, none of which were severe. The main strength of this study is the focus on solely pediatric cases, all with thorough follow-up including non-EP DWI MRI. It is the first pediatric study on the use of S53P4 BAG, providing a realistic view of the long-term cholesteatoma recidivism rate. This is also currently the largest MRI-controlled pediatric study on mastoid obliteration for cholesteatoma in general.

A population-based study published by Møller et al. of 107 children with CWU + MO surgery estimated a 5-year recidivism rate of 44% [21]. However, while Møller et al. did have long-term follow-up, no non-EP DWI MRI was used to assess disease recidivism. Few pediatric cholesteatoma studies with MRI controlled follow-up have been conducted, as most older studies did not yet have this possibility [16, 22–24]. All of our residual cholesteatoma cases and most of our recurrent cholesteatoma cases were detected using non-EP DWI MRI and likely would have been missed by otoscopic evaluation alone. This shows the necessity to control cholesteatoma patients using non-EP DWI MRI [6, 25].

Van Dinther et al., using non-EP DWI MRI to detect recidivism, showed low recidivism rates of 9% (3% recurrent and 6% residual disease) five years following surgery[26]. However, this was a small cohort of only 34 cases that were all operated by a single expert surgeon in a tertiary referral center using a CWU + MO procedure. An earlier study from the same hospital as van Dinther et al. with 47 pediatric cholesteatoma patients showed a recidivism rate of 17.3% (1.9% recurrent and 15.4% residual disease) after CWU + MO procedures [27]. In this second study, only 31 patients were MRI controlled and scans were only made at 1 and 5 years following surgery. As only 21 patients in total had a follow-up of 5 years it is possible that few would have had their 5-year MRI. Therefore, 17.3% is possibly underestimating the recidivism rate. A third study, conducted by Hellingman et al., reported MRI controlled follow-up of 25 pediatric cases as a subset of a larger study population. In this study, the authors found a 24% recidivism rate (16% recurrent and 8% residual disease) without a Kaplan–Meier curve to estimate the 5-year recidivism rate [28].

Five recidivisms were detected in the first year, six in the second, six in the third and four in the fourth and fifth years together. Therefore, we recommend non-EP DWI MRI in pediatric cases at 1, 2, 3 and 5 years following surgery, which is in line with our national guideline [29]. Importantly, the non-EP DWI MRI scans were mentally and physically well-tolerated by our patients. No general anesthesia was necessary to create the scan.

Since the non-EP DWI MRI is more sensitive than otoscopy or CT-scan for detecting cholesteatoma recidivism, it is to be expected that our recidivism rate would be high. However, it is currently unknown if all our recidivisms would have become clinically relevant. Further research is needed to evaluate the clinical relevancy of the detected cholesteatomas. Fortunately, the size of the recidivisms was limited to ≤ 1 cm in 82% of the cases. Whilst the recidivism cases of course still required surgery with general anesthesia, the revision surgeries were in general not extensive from a surgical perspective.

The residual and recurrence rate were lower in the CWU + MO group than in the CWD + MO group, although this difference was not significant. It could represent that in selected cohorts, with limited disease and sufficient overview, surgeons can choose to perform a CWU + MO procedure. However, selection bias likely played a role as the surgeon decided the surgical approach intraoperatively, based on his own preference and the extension of the cholesteatoma to poor accessible areas, such as the sinus tympani and oval window.

Cholesteatoma in children tend to have different clinical behaviors when compared to cholesteatoma in adults [2]. The nature of the disease is more aggressive, more extensive, and frequently reoccurring. Possible explanation for this difference could be Eustachian tube dysfunction (ETD), as the anatomy is suboptimal in children [2]. ETD could result in lower middle ear pressure and therefore retraction of the tympanic membrane, which is one of the leading theories on the development of cholesteatoma [30]. Other hypotheses suggest there is a causal relationship between middle ear inflammation and cholesteatoma. Palva et al. concluded that childhood middle ear inflammation results in arrest of the pneumatization of the mastoid and middle ear [31]. The underdeveloped mastoid and recurrent infections create unfavorable middle ear conditions and subsequent cholesteatoma development [32]. The effect of inflammation on the mucosa is also a driving factor in the loss of gas from the middle ear and subsequent development of atelectasis and cholesteatoma. Indeed, in our study we found that the majority of patients had a history of middle ear infections. In addition, there was an association between a history of otorrhea and residual cholesteatoma. It should be noted that this association was only present in univariate analyses and was not corrected for confounders. In addition, numbers are small and other important factors might not have been considered. The fact that 20% of our recidivism cases would develop a second recidivism further underlines the unfavorable condition of the middle ear. It also reflects the aggressiveness of the disease.

We can only speculate that a possible treatment strategy would be to intervene at an earlier moment before the initial cholesteatoma development. For example, by performing more cartilage reinforcements of the tympanic membrane or tympanomastoidectomies in patients with severe atelectasis. Cartilage reinforcement is in line with a suggestion by Jackler et al. to render the tympanic membrane nonpliable using cartilage, especially in the posterosuperior area [32]. The cartilage reinforcement should subsequently inhibit cholesteatoma development. However, some of our patients had cartilage reinforcement of the tympanic membrane and developed a cholesteatoma anyway.

Since our recidivism rate is still substantial, we also should explore further options to reduce recidivism rates. The first is obliteration of the epitympanum. In 2015–2016, we started systematically obliterating the epitympanum, as most recidivisms would develop in that area. In our cohort, six of the cholesteatoma recurrences and 5 of the residual cholesteatomas developed in the epitympanum. This shows the importance adequately exploring and obliterating of the epitympanum. Furthermore, pediatric cholesteatoma is fairly rare, e.g., in our department only 65 cases were operated in nine years by four surgeons. Therefore, another possible improvement could be the clustering of the pediatric cholesteatoma surgeries to two otologists, ensuring the highest degree of expertise. Given the rarity of pediatric cholesteatomas and the high recurrence rate, it is likely that clustering of pediatric cholesteatoma surgery can improve the outcome. A similar suggestion was made by Soldati et al., who presented that a surgeon should have operated at least 350 cases before achieving satisfying results in children [33]. Both suggestions could improve the outcomes, but cholesteatoma recidivism will still occur. Counseling of patients and parents will remain crucial for them to understand the recidivistic nature of the disease.

A significant improvement in the ABG was observed in patients with a TORP. The postoperative ABG did not significantly differ between the PORP and TORP group, showing that the hearing outcome was comparable between these two groups. Therefore, acceptable hearing outcome is still possible in cases with more severe deterioration of the ossicular chain. However, closure of the ABG was only achieved in a small subset of patients, which is in line with other publications [22, 34]. This could potentially be a matter of concern, as adequate hearing is essential for the school performance of children [35]. Hearing aids are fortunately an option and more research is required to investigate this potential cause for concern. Counseling of patients and parents on the expected hearing outcome will continue to play an important role.

There are some limitations to be addressed: first, the retrospective character of this study did not allow for the further classification of the cholesteatomas with systems, such as STAMCO or CHOLE. The necessary detailed information required for staging was frequently not present in the operation report. As a result of this, it could be more difficult to compare our research to other studies. Cholesteatoma classification may also be prognostically relevant, although this has recently been challenged by Eggink et al. [36, 37]. Second, we did not compare our results with a cohort without obliteration due to differences in follow-up protocols in our center. Our surgical technique changed in 2011. Patients from before 2011, who received a mastoidectomy without obliteration, did not yet receive standardized follow-up with non-EP DWI MRI. Therefore, our study does not show superiority of mastoid obliteration over non-obliterative techniques. Unfortunately, direct comparison is also missing in other studies and hence both options are still justifiable. Third, follow-up time in our cohort was not homogenous. However, since the mean follow-up period was 58 months and we also used a Kaplan–Meier curve to estimate the 5-year recidivism rate, we feel that this has not significantly influenced our results.

In conclusion, our results show long-term safety of S53P4 BAG for mastoid obliteration in pediatric cholesteatoma surgery. Few complications were seen and a dry ear was achieved in almost all patients. The sensitive non-invasive non-EP DWI MRI was used to assess cholesteatoma recidivisms, which was well-tolerated by all patients. Recidivism rates for pediatric cholesteatoma are still substantial even after mastoid obliteration. Future research should focus on additional treatment strategies, such as adjuvant treatment or earlier interventions, and predictive factors for recidivism.

## Supplementary Information

Below is the link to the electronic supplementary material.

Supplementary file1 (DOCX 22 KB)
