# Supplementary material for: Cholesteatoma surgery in the pediatric population: remaining challenges in the era of mastoid obliteration
Source: Eur Arch Otorhinolaryngol. 2022 Oct 8;280(4):1713–22. doi: 10.1007/s00405-022-07669-0 (PMC9988747; doi:10.1007/s00405-022-07669-0)
Supplement: Supplementary file 1 — Supplementary file1 (DOCX 22 KB) [file 405_2022_7669_MOESM1_ESM.docx]

| Supplementary Table 1. Characteristics of recurrent and residual cases | | | | | | | | | | | | |
| --- | --- | --- | --- | --- | --- | --- | --- | --- | --- | --- | --- | --- |
| Case # | Sex, age (years) | Previous surgery for cholesteatoma | Type of surgery | ABG after primary surgery (dB) | Time to recidivism (months) | Merchant grade before revision | Recurrent / Residual | Location | Detection method | Size recidivism on MRI (mm) | Type of revision surgery | Recidivism free period after revision (months) |
|  | F,15 | Yes | CWU | 36,25 | 8 | 1 | Residual | Epitympanum | MRI | 9 | CWD | n.a. (second recidivism) |
|  | M, 14 | No | CWU | 12,5 | 59 | 0 | Residual | Epitympanum | MRI | 6 | CWU | 54 |
|  | M, 10 | No | CWU | 53,75 | 10 | 0 | Residual | Epitympanum | MRI | 7 | CWD | n.a. (second recidivism) |
|  | M, 13 | Yes | CWD | 11,25 | 26 | 0 | Recurrent | Epitympanum | Otoscopy | na | Endoscopic transcanal | 56 |
|  | F, 11 | No | CWD | 56,25 | 24 | 0 | Residual | Tympanic sinus | MRI | 7 | CWD | n.a. (second recidivism) |
|  | M, 8 | Yes | CWD | 42,25 | 28 | 0 | Residual | Epitympanum | MRI | 5 | CWD | 48 |
|  | M, 7 | No | CWD | 28,75 | 25 | 0 | Recurrent | Epitympanum | Otoscopy | na | CWD | 54 |
|  | F, 10 | Yes | CWD | n.a. | 12 | 0 | Recurrent | Mesotympanum | Otoscopy | na | CWD | n.a. (second recidivism) |
|  | M, 12 | No | CWD | n.a. | 44 | 1 | Recurrent | Epitympanum | MRI | 7 | CWD | 28 |
|  | M, 13 | No | CWD | 60 | 25 | 1 | Residual | Tympanic sinus | MRI | 6 | CWD | 42 |
|  | M, 8 | No | CWD | 26,25 | 12 | 2 | Residual | Stapes footplate | MRI | 6 | CWD | 60 |
|  | F, 16 | No | CWD | 23,75 | 16 | 0 | Recurrent | Epitympanum | MRI | 7 | Endoscopic transcanal | 25 |
|  | M, 16 | No | CWD | 21,75 | 12 | 0 | Residual | Epitympanum | MRI | 8 | CWD | 40 |
|  | M, 8 | No | CWD | n.a. | 57 | 2 | Recurrent | Mastoid | MRI | 15 | CWD | 0 |
|  | F, 7 | No | CWD | 37,5 | 26 | 0 | Residual | Stapes footplate | MRI | na | Endoscopic transcanal | 29 |
|  | M, 13 | No | CWD | n.a | 38 | 0 | Recurrent | Epitympanum | MRI | 23 | CWD | 12 |
|  | M, 6 | No | CWD | 53,75 | 25 | 0 | Residual | Stapes footplate | MRI | 8 | CWD | 12 |
|  | M, 11 | No | CWD | 25 | 14 | 1 | Residual | Stapes footplate | MRI | 12 | CWD | 16 |
|  | M, 9 | No | CWD | 17,5 | 19 | 0 | Recurrent | Epitympanum | MRI | 10 | Endoscopic transcanal | 5 |
|  | F, 17 | No | CWD | 28,75 | 14 | 0 | Residual | Tympanic sinus | MRI | 3 | Endoscopic transcanal | 5 |
